# Public Spaces as Knowledgescapes: Understanding the Relationship between the Built Environment and Creative Encounters at Dutch University Campuses and Science Parks

**DOI:** 10.3390/ijerph17207421

**Published:** 2020-10-12

**Authors:** Isabelle Soares, Gerd Weitkamp, Claudia Yamu

**Affiliations:** 1 Department of Spatial Planning and Environment, Faculty of Spatial Sciences, University of Groningen, 9747AJ Groningen, The Netherlands; claudia.yamu@rug.nl; 2 Department of Cultural Geography, Faculty of Spatial Sciences, University of Groningen, 9747AJ Groningen, The Netherlands; s.g.weitkamp@rug.nl

**Keywords:** volunteered geographic information (VGI), public participatory geographic information system (PPGIS), spatial affordances for creativity, university campus, science park, public space, urban design

## Abstract

The success of university campuses depends on the interrelations between creative encounters and the built environment, conceptualised here as spatial affordances for creativity. Such an interface plays a fundamental role in interactions for knowledge sharing and the exchange of ideas on campus. Due to campus public spaces generally being considered as the leftovers between buildings and classrooms, undermanaged, and overlooked, little is known about the extent to which this built environment enables or inhibits creative encounters in such spaces. The inner-city campuses and science parks (SPs) of Amsterdam and Utrecht, the case-studies of this research, differ in terms of their location relative to the city, their masterplan typologies and the arrangement of buildings. However, they are similar in terms of the aforementioned issues of public spaces. The novelty of this research is the attempt to overcome such issues using an innovative mixed-methods approach that tests the ‘spatial affordances for creativity’ with empirical data collection and analysis. This raises the importance of mapping, quantifying and analysing the spatial distribution of momentary perceptions, experiences, and feelings of people with methods such as volunteered geographic information (VGI). The results show that proximity between multiple urban functions and physical features, such as parks, cafés and urban seating are important when it comes to explaining the high frequency of creative encounters between people. Urban designers of campuses can use the applied method as a tool to plan and design attractive public spaces that provide creativity through the transfer of tacit knowledge, social well-being, positive momentary perceptions, sense of community, and a sense of place.

## 1. Introduction 

University campuses are vital actors in the global knowledge economy, central players in emergent innovation systems and active agents that can play a driving role in the innovation process and commercialization of knowledge [1,2,3]. As suggested by Glaeser [4], the co-presence of educated individuals in one location is linked to new ideas, creativity and, consequently, long-term economic growth. Public spaces at university campuses are important knowledge and creative hubs and enablers of creativity as well as social well-being through a sense of place and community [5,6,7]. Past studies have identified positive associations between creativity and well-being [8], suggesting that public spaces that afford social interactions and social capital are associated with improved social well-being [9,10]. In the context of campuses, such spaces are catalysts that bring people, ideas and resources together before a creative process can occur [11,12]. There is a heightened interest within the urban design and planning field in understanding how the built environment, and more specifically (campus) public spaces, play a role in creativity through knowledge sharing and the exchange of ideas [13,14]. The interface between the built environment and the actions and reactions that it evokes in people can be described as ‘spatial affordances for creativity’. We understand that creativity emerges and develops in dynamic interaction between the individual and their spatial environment [12,15,16,17,18]. 

Spatial affordances for creativity is still an emerging field. The term was first used by Sailer [19], who found a relationship between the physical features of office spaces and people’s contacts which tacit knowledge is shared. It was defined as the ‘ability to engage in creative work depending on the affordances of the spatial layout’ [19] (p. 9). This is in line with James Gibson’s [20] theory of affordances, defined as the relations between the abilities of organisms (humans) and features of the environment [20,21]. Such relations refer to sets of functional, social and emotional opportunities emerging from social, spatial and immaterial characteristics of the environment [22,23]. The concept of affordances highlights several interrelated qualities of environments [24] and the action-oriented aspects of human perceptions [25]. Spatial affordances for creativity thus relates to peoples’ well-being, since participation and integration in the immediate social environment are important to both mental and physical health [8,26,27,28].

And yet, spatial affordances of university campuses for creativity have been the focus of relatively limited theoretical and empirical research. A broad theoretical framework was established to show how creativity enables the spatial configuration of cities, campuses and city-campus relationships [3,29] and how space, sense of place and perceptions are important for enabling creativity [12,30]. Empirical research on campus planning and design explored relationships between cities and campuses in terms of accessibility [31,32], how the design of a campus masterplan can retain students or not [33,34], placemaking in public spaces connected to knowledge formation and the university-industry-government (triple-helix) relationship [35], and the interrelationship between creativity, interaction patterns and physical space in office environments [19,36,37]. Empirical studies on public participatory geographic information system (PPGIS) have addressed affordances by mapping and quantifying the spatial distribution of social values, perceptions, preferences and other attributes using a variety of spatial techniques [38,39,40,41]. 

Given the above, there is a lack of empirical research on and analysis of spatial affordances for creativity in public spaces on a university campus. Although the planning and design of campuses are the primary ways in which a university articulates its vision of future development [42], public spaces are often undermanaged and overlooked, and seem to be leftovers between classrooms, buildings, car parks, and bicycle lanes. This can be attributed to fragmented governance on the part of multiple campus actors [43,44], which has implications for the quality of campus spaces. A mixed-methods approach involving participatory methods, such as PPGIS, can therefore be an effective tool for the urban design of public spaces, since it can generate detailed information on spatial and perceptual aspects of creativity [45]. This approach was applied to our case studies of the inner-city university campuses and science parks (SPs) in the cities of Amsterdam and Utrecht in the Netherlands. 

We address the research gap by understanding patterns of human perceptions in public spaces and how the built environment plays a role in those patterns, using the concept of spatial affordances for creativity [12,20,21,24]. We answer the following research questions: (1) Which public spaces do people perceive as affording creativity through knowledge sharing and the exchange of ideas? (2) How does the presence, absence, and proximity to spatial features of the built environment play a role in public spaces with a high frequency of creative encounters? (3) How do open and semi-public spaces that afford a high frequency of creative encounters differ between inner-city campuses and SPs? 

To answer the research questions, this research applied an innovative mixed methodology to test the ‘spatial affordances for creativity’ theoretical framework with empirical data collection and analysis. The method consisted of (1) data collection and analysis of volunteered geographic information (VGI), a type of PPGIS, which represents ‘patches’ of intensities of human perceptions, (2) integration of these geo-referenced perceptual data with quantification of the built environment (urban functions and physical features), operationalized through proximity analyses (this analysis highlights how creative encounters are affected by the built environment), and (3) a content analysis of photography of public spaces, pointing to differences and similarities between the knowledgescapes of the case studies. The main contribution of this paper is the mixed-methods approach, which provides insights for future campus planning and design by addressing the aforementioned research gap and research problem, as it takes into consideration what users understand and perceive as spatial affordances for creativity. 

The remainder of the paper is structured as follows: Section 2 presents a literature review, addressing spatial affordances for creativity, campus planning and design, public spaces, human perceptions and sense of place. Section 3 describes the data collection and methodology, followed by a discussion of the main results. The final section contains the conclusion, limitations and recommendations for future work.

## 2. Literature Review 

### 2.1. Spatial Affordances for Creativity of University Campuses

The interrelations between creative encounters and characteristics of the built environment are described here as ‘spatial affordances for creativity’ [19]. Creativity is defined as ‘the ability to come up with ideas or artefacts that are surprising and valuable’ [46] (p. 1). Creativity is not an innate attribute of a single individual, no matter how intelligent and talented that person might be. A stimulating knowledge environment and a talented individual must come together and interact before a creative process can occur [11,12,15,16]. Therefore, spatial affordances for creativity relates to what the environment has to ‘offer’ a (creative) individual and how it guides and facilitates, and also constrains, knowledge sharing and the exchange of ideas [25]. Such affordances are sustained when different characteristics of the built environment affect an individual’s physical abilities, emotions, intentions and meaningful relations [45,47]. For instance, the affordance of a ‘walk-on-able’ surface refers to a person’s relationship with an environmental feature (sidewalk). This is in line with Helbrecht [48], who states that ‘the look and feel of an urban landscape can play a role in knowledge production processes’ [48] (p. 197). 

The success of a university campus derives from causal interactions that facilitate the generation, diffusion and application of creativity [30]. Campus public spaces are therefore expected to afford face-to-face interactions and the constant transfer of tacit knowledge [49]. Matthiesen [50] coined such public spaces knowledgescapes. These are meeting points that promote interplay between formal and informal interaction networks [12,50]. To share knowledge, people need to experience a sense of community and attachment to a certain place. A strong sense of place has been associated with improved social well-being [10], trust, and consequently the exchange of information between people [8,51]. 

Place is characterized by a site or spot where a person performs an activity, faces a challenge or perceives stimuli and clues [7,52,53,54]. According to Canter, ‘a place is the result of relationships between actions, conceptions, and physical attributes’ [55] (p. 159). For university campuses, places are social spaces and nodes of knowledge generation and distribution [30,56]. People are emotionally attached to places, have preferences for certain places or gather regularly at specific places for various functional, symbolic and emotional reasons [57]. To achieve vital and thriving places, perception plays a fundamental role in people’s experiences connected to a sense of place. People require perceptual means to guide their actions and to support exploration, sometimes over considerable distances. Heft [24] explains that perceiving and acting are fundamental to sustaining life in nearly all complex organisms. Consequently, spatial affordances for creativity depend on the interface between perceptions and the built environment, resulting in a person’s action. This also holds true for public spaces on a university campus.

### 2.2. Public Spaces: The Interface between Perceptions and Built Environment Features

Public spaces are conceptualized as spaces accessible to all groups, the main stage of urban life, facilitating encounters and the exchange of experiences and fostering a tolerant urban society through exposure to different people and their traditions [58,59,60,61,62,63,64]. The public spaces of inner-city campuses and SPs have two main typologies: open and semi-public spaces. Open public spaces are defined as ‘open, publicly accessible places where people go for group or individual activities’ [59] (p. 50) and encompass many different types, such as parks, plazas, streets and sidewalks [59]. For semi-public spaces, we use Oldenburg’s definition of ‘third places’, which host ‘regular, voluntary, informal and happily anticipated gatherings of individuals beyond the realms of home and work’ [61] (p. 16). They are community gathering places mostly accessible by people who work and study at a campus, such as shared spaces, canteens, cafés, bars and restaurants [58]. 

Scholars such as Whyte [65], Dober [66,67], Strange and Banning [68,69], Kenney et al. [70], Hajrasouliha [33,34], Wood and Dovey [14], Francis et al. [10] Lau and Yang [71] and Zeng et al. [72] suggest that the following characteristics of the built environment must be considered in the planning and design of successful knowledgescapes:

(1) For the organization of urban functions, a campus has to provide a mix of sports, research, residential and various academic activities.
(2) Public spaces are reinforced by the appropriate proximity of buildings (density) and the juxtaposition of activities that complement one another (diversity).
(3) Open and semi-public, indoor and outdoor social spaces should be scattered throughout the overall framework of the campus (not just at the campus centre), such as lounges in the halls of residence, meeting spaces in the lobbies of buildings, and outdoor sitting areas. This guarantees informal settings that facilitate interactions, including sitting opportunities, trees, shade and shelter, cafés or dining halls in various places on campus.
(4) The availability of natural features, such as water and green areas, may improve one’s mental status through creating opportunities for recreation and relaxation, and promoting social well-being as well as positive physical and mental health benefits.

Overall, this suggests that proximity between diverse urban functions and physical features within a campus can enhance creative encounters in public spaces. Proximity refers to the evaluation of the ability to reach urban places, and the quantity and quality of places that can be accessed [73]. Assessing distances between mapped perceptions of places that enable creativity and characteristics of the built environment can be a beneficial tool for proposing design interventions that would help facilitate creativity through knowledge sharing and the exchange of ideas at university campuses. 

## 3. Materials and Methods 

### 3.1. Case Studies

**Figure 1:**
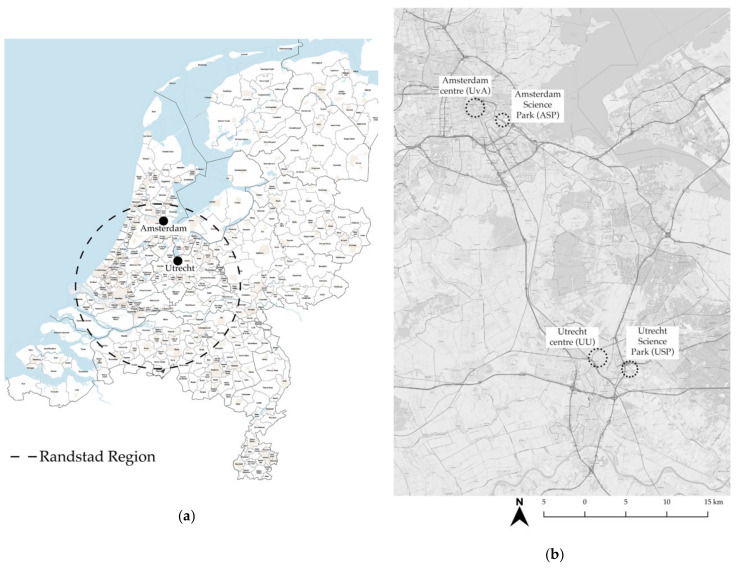
Amsterdam and Utrecht: (**a**) Location in the Netherlands [77]; (**b**) Amsterdam and Utrecht inner-city campuses and SPs in the context of the Randstad.

This study empirically investigates two types of university campus located in two Dutch cities. Both Amsterdam and Utrecht are located in the Randstad region of the Netherlands and feature the two campus types that are the subject of this research: the ‘dependent urban fabric’ campus, which we call inner-city campus, and the ‘autonomous urban fabric’ campus, referred to here as science parks (SP) [74,75,76]. Both cities (Amsterdam and Utrecht) and their campuses are similar in terms of how they were developed and implemented. Inner-city campuses have evolved with the old city centre and SPs were implemented in isolated areas on the city peripheries. Figure 1 shows the location of the two cities and campuses. 

The definition of ‘campus’ deserves a brief explanation in the context of the Netherlands because the Anglo-Saxon model is readily associated with a fairly isolated area, inside or outside the city. For this research, we use the definitions of den Heijer et al. [78], in which the term campus includes all buildings and sites that are part of the university or are used for university-related functions, or which are used, rented or owned by the university. University campus also refers to a collection of buildings scattered throughout a city’s main centre. 

**Figure 2:**
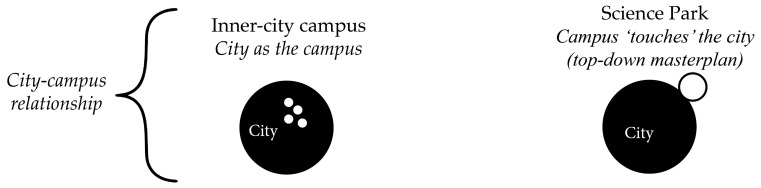
City-university spatial relationships. City (black circle) and campus (white circles).

**Figure 3:**
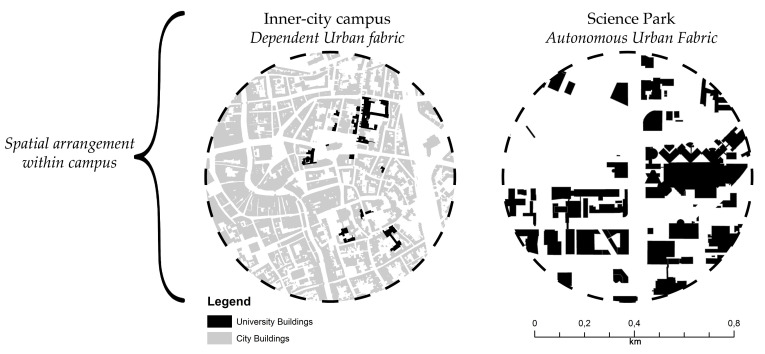
Buildings and public spaces: built (black and grey) and unbuilt (white).

Figure 2 and Figure 3 show the campus-city spatial relationships and the masterplan typologies and public spaces of Dutch campuses, based on Hajrasouliha [33], den Heijer and Magdaniel [74], and da Silva and Heitor [75]. The inner-city campus is characterized by faculty buildings that have grown together spatially with the city, as a mix of ‘town and gown’. They are ensembles of buildings of different styles and ages that coalesced to form a mutual spatial-economic relationship with the host city [76,79]. There is no clear demarcation in the form of a campus masterplan, but rather a sum of independent buildings located in geographic proximity [75,80]. The buildings are usually no larger than a city block and are constantly being adapted to make room for emergent academic activities and a growing number of students [81,82]. 

The ‘autonomous urban fabric’ campus emerged with the creation of new disciplines such as the natural sciences and technology after World War II. These campuses were designed as a top-down masterplan, implemented in the city suburbs that were generally accessible by car and public transport. Since the 1990s, they evolved from university initiatives to triple-helix environments with partnerships between university, industry and governments [83,84], and acquired the name science parks [85]. Their mix of buildings includes multi-tenant university buildings (including incubators specifically aimed at start-ups), collaboration spaces, single-tenant buildings, student housing and empty plots for future development [84]. 

The public spaces of inner-city campuses and SPs also have differences and similarities. The public spaces of the inner-city campuses are predominantly roads, sidewalks and semi-public spaces, such as enclosed gardens and patios with limited access. In this context, building façades commonly face public urban squares, which have a strong relationship with the streets of the old city center [79]. Notably, the public spaces of SPs are spatially and socially disconnected from their immediate context, as are their buildings [33,86,87]. They are designed as independent entities, with buildings and public spaces acting as free-standing objects along a strictly orthogonal grid of roads between green fields [74]. 

#### 3.1.1. Amsterdam Inner-City Campus and Science Park 

**Figure 4:**
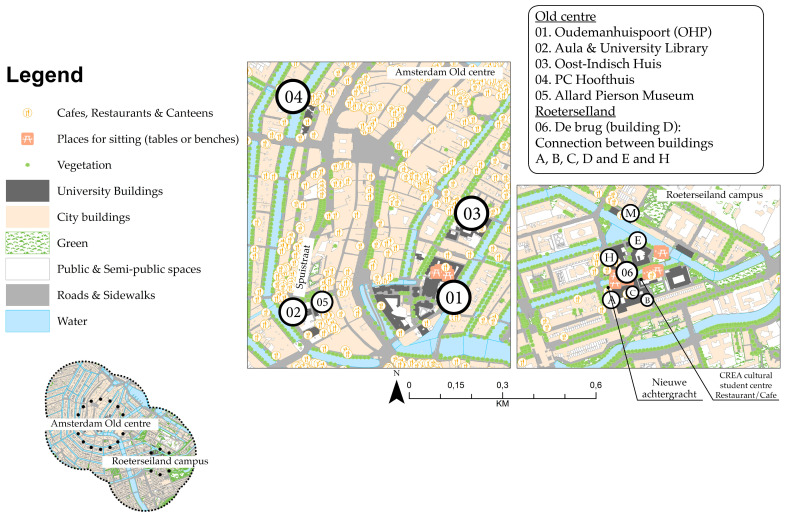
Amsterdam inner-city campuses.

The city of Amsterdam has two inner-city campuses and one SP, which are part of the University of Amsterdam (UvA). The Oudemanshuisport (OHP), Oost-indisch huis and PC Hoofthuis buildings are located in the old centre and are part of the Faculty of Humanities and university support services (Figure 4). The Roeterseiland (RI) campus is characterized by a top-down building complex that contrasts with the organic structure of the inner city [88]. Since a major renovation, completed in 2017, RI has housed the faculties of Economics and Business, Social and Behavioural Sciences and Law. 

**Figure 5:**
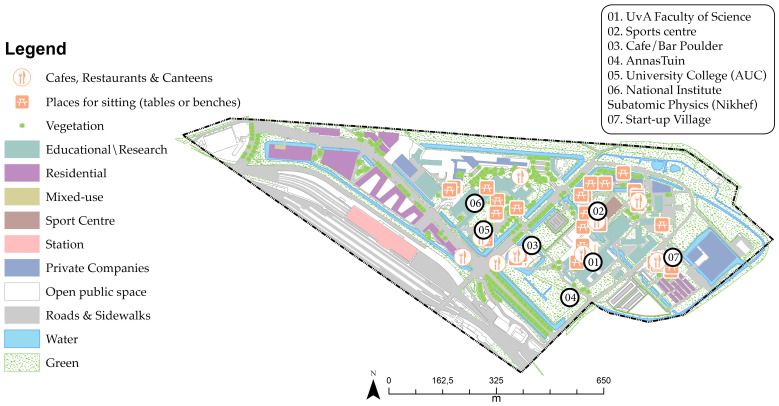
Land-use at Amsterdam Science Park (ASP).

Amsterdam Science Park (ASP), located in Amsterdam’s south-east, was first established in 1946. Since then, multiple research and educational institutions focusing on technology and the natural sciences have been implemented across the campus. In the early nineties, companies were implemented at the campus through the Matrix innovation center. In 2012, Amsterdam University College (AUC) was established, together with student housing [89]. ASP is also home to the Universum sports centre, Anna’s Tuin (a community garden) and a start-up village (Figure 5). 

#### 3.1.2. Utrecht Inner-City Campus and Science Park

**Figure 6:**
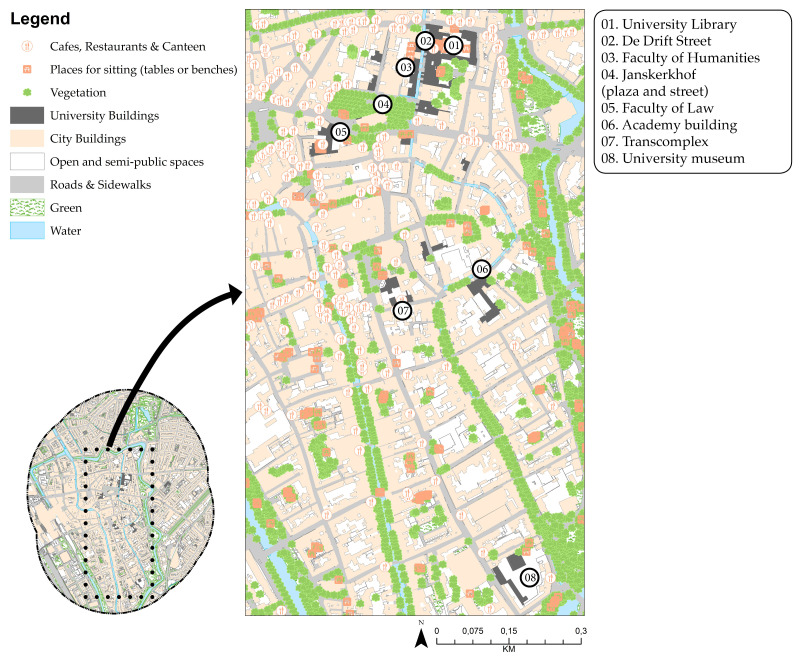
Utrecht inner-city campus.

Like the OHP in Amsterdam, the inner-city campus of Utrecht University (UU) is composed of buildings for the social and economic sciences, which are scattered throughout the old city centre, for example along Drift and Janskerkhof streets (Figure 6). Many national monuments also make up this campus: the academy building, university museum and university library [90].

**Figure 7:**
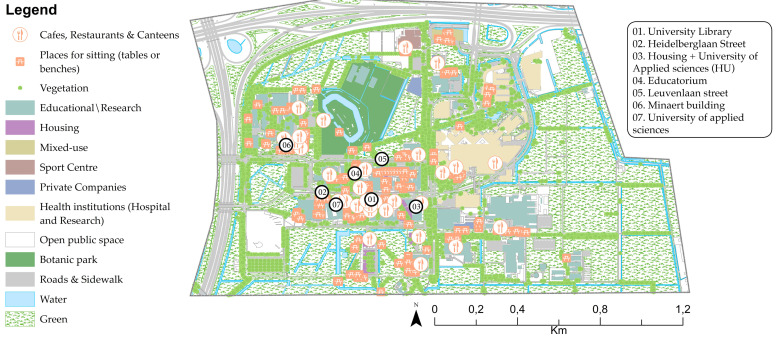
Land use at Utrecht Science Park (USP).

Utrecht Science Park (USP), formerly called the Uithof, is located on the eastern edge of the city of Utrecht, near the Botanic Gardens, which were established in 1639, three years after the foundation of the UU [91]. Centuries later, in the 1960s, the university campus was established to house the veterinary school. The campus hosts seven faculties of the UU (Science, Veterinary Medicine, Medicine, Geosciences, and Social and Behavioural Sciences), student accommodation, the main library, sporting facilities, food outlets and shops [92,93]. It is also home to University of Applied Sciences Utrecht (HU). Health-based institutes and companies are located at USP because of the proximity to the UMC (Figure 7).

### 3.2. Volunteered Geographic Information (VGI) and Spatial Analysis

Data were collected using VGI, a type of PPGIS [38] that combines internet maps with traditional questionnaires [41,94]. Such methods have been used in diverse place-based studies, examining for example the relationship between design characteristics and social qualities relating to the perceived safety of inhabitants of a neighbourhood [95], the application of PPGIS for planning urban green infrastructure [96], multiple-level influences of health behaviours and factors that influence active travel by older adults [94], the combination of accessibility analysis with PPGIS of users’ environmental perceptions [97] and the VGI of locations that enhance relaxation and reduce stress among university students [98]. Although perceptions and objective properties of places can be mapped and quantified with PPGIS and VGI [38,99], such methods had not yet been used to explore perceptions and experiences of creative encounters at university campuses and SPs.

**Figure 8:**
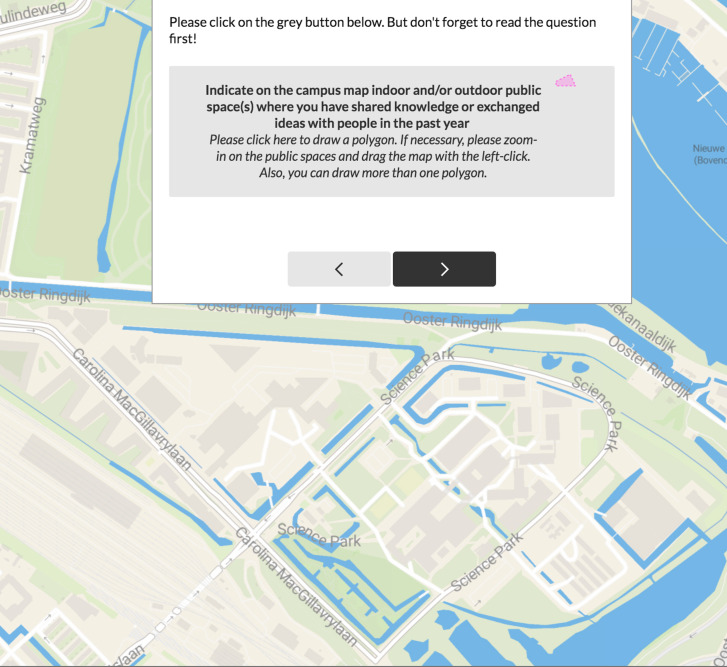
The online interface of the survey.

For the present study, perceptual data were collected through the urban-focused web platform Maptionnaire (Mapita, Helsinki, Finland) [100,101]. The survey was designed to empirically record spatial clusters of human perceptions by identifying public spaces that afford creativity. Creativity is represented here by the act of sharing knowledge and exchanging ideas with others. In accordance with this, participants were asked to draw polygons on the campus map to indicate the indoor and/or outdoor public spaces where they shared knowledge and exchanged ideas with people in the past year (Figure 8).

In total, 318 people answered the questionnaire, generating 511 polygon responses. The data were collected from students, university employees, science park company employees and visitors, in September and October 2019. Even though we collected people’s socio-demographic data (Table 1), the focus of this research is on the landscape ‘patches’ of human perceptions throughout the campuses. Participants were approached on-site to answer the questionnaire, with the researchers present in case of difficulties in filling out the questionnaire. Some participants completed the questionnaire on their own at a later time. This method was developed to provide a large sample of perceptions regarding campus public spaces rather than produce a representative sample of the population as a whole.

The data collection was conducted according to the Netherlands Code of Conduct for Research Integrity (NWO) [102] and the General Data Protection Regulation (GDPR) [103]. We confirm that all participants gave their informed consent for inclusion before filling out the questionnaire. The study was conducted in accordance with the Declaration of Helsinki, and the protocol was approved by the Ethics Committee of the Faculty of Spatial Sciences of the University of Groningen. 

#### Data Preparation and Analysis

This study used VGI perceptual data that represent frequencies of usage of indoor, outdoor public and semi-public spaces where people share knowledge and exchange ideas. The collected polygon data of all participants were aggregated and summarized into 100 × 100 m grid cells, a similar approach to the one adopted by Curtis et al. [104]. The size of the grid cells is proportional to the scale of public spaces and buildings. For the perimeter of the study areas, we used institutional boundaries for SPs and inner-city campuses. The limits were set in accordance with an 800 m distance buffer equivalent to 10 min walk from university buildings. Table 2 summarizes the collected data.

High and low possibilities for sharing knowledge and exchanging ideas were found throughout the campuses using a ‘spatial join’ operation. The 100 × 100 m grid cells were intersected with the VGI data (polygons) the number of polygons mapped in each location was attributed to each cell (or campus area). The result was a new column in the attribute table with a frequency count (or ‘join count’) showing the number of objects located in each grid cell [104]. For the results, a grid-cell map was produced for each campus representing the locations where respondents had shared knowledge with people in the past year. Here, the ‘join count’ values are called ‘VGI values’. The degree of variability across the campuses is partly due to the different sizes and contexts of each study area.

### 3.3. The Interface between Mapped Perceptions and Built-Environment Features

We next explored spaces shown to have a high frequency of creative encounters, relating the grid-cell VGI values to built environment characteristics. These characteristics are divided into two classes: urban functions and physical features. The presence, absence and proximity of each 100 × 100 m grid cell to the nearest features were calculated in similar fashion to other PPGIS studies [73,97,105]. We thus followed Talen’s suggestion that ‘For urban design purposes, the best approach is to select a set of urban facilities or places that are important to have access to and then evaluate distances to them’ [73] (p. 144). To strategize the comparison between campuses, the 10 cells with the highest VGI values of each study area were selected for analysis and comparison. From now on, we will refer to these cells as ‘high-value cells’, which represent the campus knowledgescapes. Since this was our first attempt to measure the interface between mapped perceptions and data representing the built environment, we decided to attribute the same weight (i.e., importance) to all built environment characteristics.

Secondary data representing the built environment was gathered via the ArcGIS online (developer ESRI Rotterdam, The Netherlands, via UG Geoportal) [106]: (1) the Basisregistratie Grootschalige Topografie (BGT) (developed by PDOK, Amsterdam, The Netherlands), the dataset for large-scale topography [107]; (2) Top10.NL, for Dutch topographic layers (developed by PDOK, Amsterdam, The Netherlands), [108]; and (3) Open Street Map (OSM) (developed by Open Street Map Foundation, Cambridge, UK) [109]. Those datasets contain shapefiles of buildings, roads, watercourses, open and semi-public public spaces, railway lines, trees, street furniture and more. Table 3 categorizes the built-environment characteristics to be measured and analysed in terms of their presence, absence and proximity to 100 × 100 m grid cells.

### 3.4. Differences and Similarities between Public Spaces of Inner-City Campuses and Science Parks 

For the comparative analysis, we address the main differences and similarities of inner-city campuses and SPs using the results of the high-value cells. We also use photography of open public spaces located at those high-value areas to visualize and exemplify differences and similarities between the knowledgescapes of the four case study areas: campus open public spaces (streets, plazas and green areas) and semi-public spaces (indoor and outdoor areas). We developed Table 4 to summarize public space concepts and typologies and to strategize the content analysis of photography. This categorization was found useful since past research has suggested that creative encounters depend on city-university spatial relationships, as well as on the arrangement of buildings and public spaces [69].

## 4. Results and Discussion

### 4.1. Perceptions of Creative Encounters throughout Campus Public Spaces 

**Figure 9:**
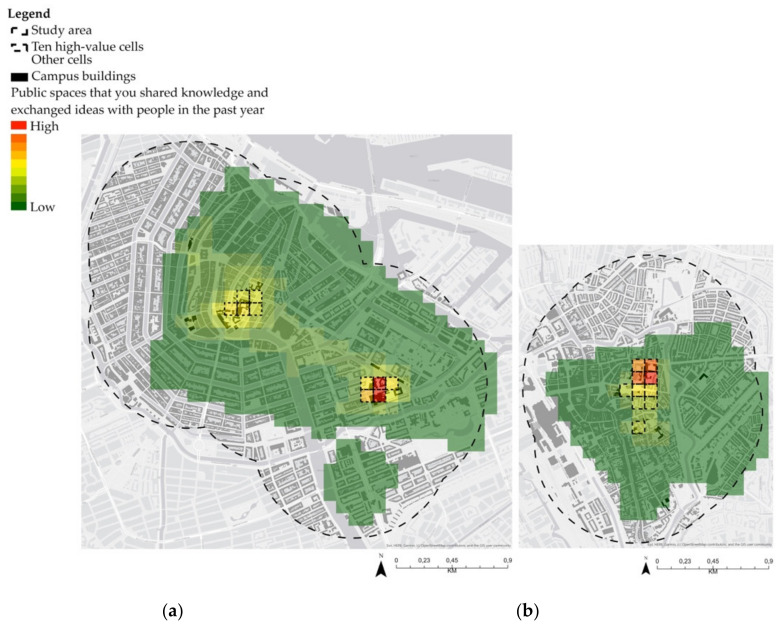
Results of VGI data for inner-city campuses: (**a**) Amsterdam; (**b**) Utrecht.

**Figure 10:**
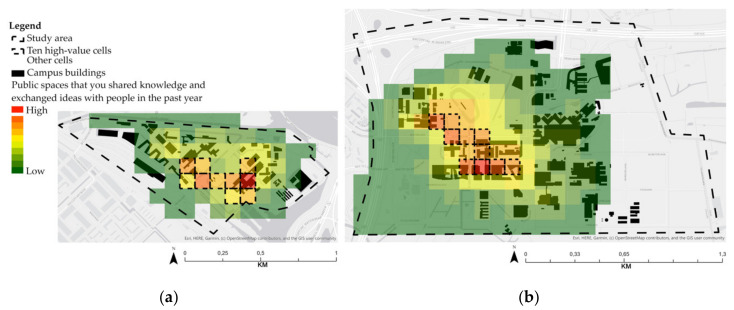
Results of VGI data for science parks: (**a**) Amsterdam; (**b**) Utrecht.

To show the rationale behind the locations identified by respondents, Figure 9 and Figure 10 present the results of the aggregated 100 × 100 m grid cells. Results are represented graphically using a colour scale ranging from red, referring to spaces with a high frequency of creativity through knowledge sharing and exchange of ideas, to dark green, representing low encounters. Table 5 illustrates the percentage of answers within the highest cell of each campus. This shows that the majority of responses for high creativity potential are concentrated in a few locations. 

For inner-city campuses, results show that the further away from university buildings, the smaller the chances of creative encounters (Figure 9). For instance, at the Utrecht inner-city campus, 70.6% of the responses are located in one cell. In contrast, the high-value cells at SPs are spread throughout the campuses, distributed along diverse academic and non-academic activities (Figure 10). In this context, more than one cluster or location has high potential for creativity.

### 4.2. The Proximity between High-Value Cells and Characteristics of the Built Environment 

#### 4.2.1. Amsterdam Inner-City Campuses and Science Park

**Figure 11:**
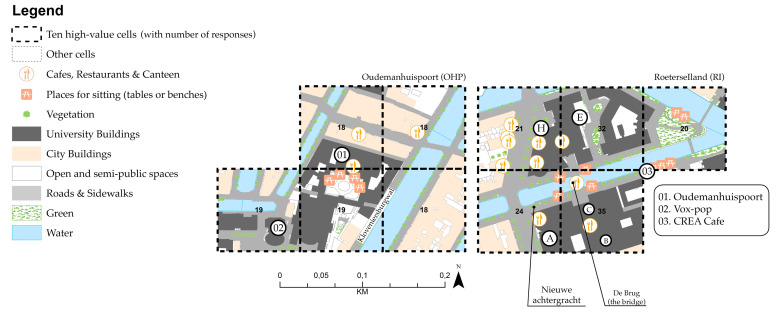
Ten high-value cells at Amsterdam inner-city campuses: UvA OHP (**left**) and UvA RI (**right**).

For Amsterdam inner-city campuses, the results of the ten high-value cells are located in the OHP building and RI buildings complex. Figure 11 shows that places that afford creative encounters depend on the proximity to various built-environment features such as university buildings (with classrooms and places to study), cafés, canteens and places to sit between classes and other engagements (Table 6). The main difference between Amsterdam inner-city campuses is the diversity of disciplines. RI’s high-value cells are located across multiple faculties. For instance, cell 97 (*n* = 35) is located between the ABC and HE buildings, representing indoor and outdoor public spaces showing a high frequency of creative encounters. At the OHP, the cell with the highest value (*n* = 19) depicts indoor and outdoor areas of only one building (faculty of humanities). Table 6 shows that all fundamental urban functions and physical features are present or close to the high-value cells. 

**Figure 12:**
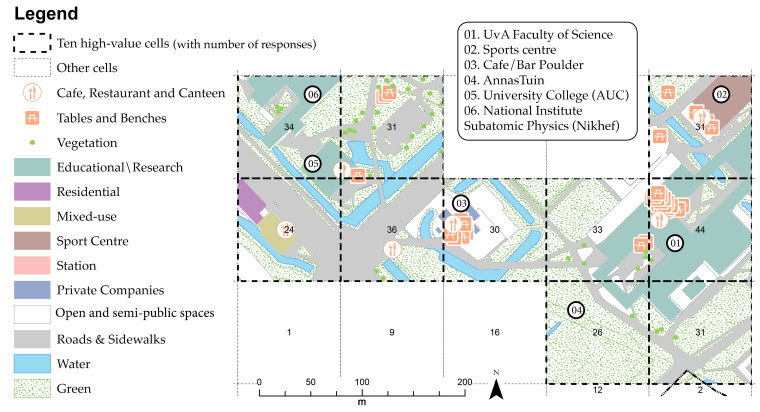
Ten high-value cells at ASP.

Results show that the knowledgescapes of ASP are function-oriented, confirming that proximity between built-environment features does enable creativity (Figure 12 and Table 7). For instance, the cell located at the main university building (*n* = 44) had the highest frequency of encounters of the entire campus. Additionally, the cells located at the Anna’s Tuin community garden (*n* = 26) and the only campus pub (Poulder) (*n* = 30) show a high frequency of knowledge sharing and exchange of ideas, and therefore high creativity. In short, Table 7 confirms that all fundamental urban functions and physical features are present or close to the ASP high-value cells. 

#### 4.2.2. Utrecht Inner-City Campus and Science Park 

**Figure 13:**
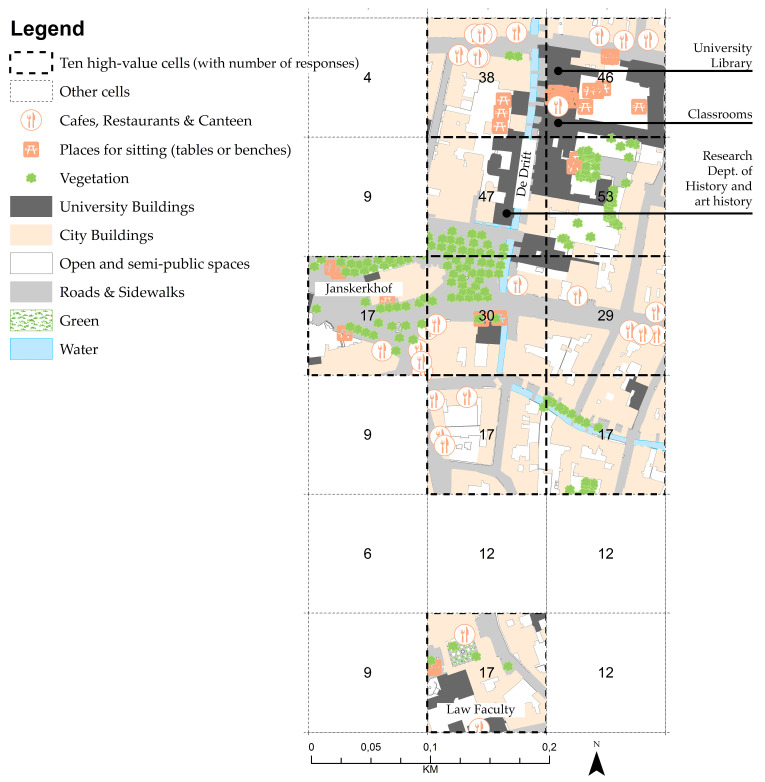
Ten high-value cells at Utrecht University (UU) inner-city campus.

For the Utrecht inner-city campus, the ten high-value cells are located within walking distance of the University library (*n* = 46), classrooms and research buildings located at Drift street (*n* = 53 and *n* = 47), as shown in Figure 13 and Table 8. As with the Amsterdam inner-city campus, all fundamental built-environment features are present or close to the high-value cells at the Utrecht inner-city campus. 

**Figure 14:**
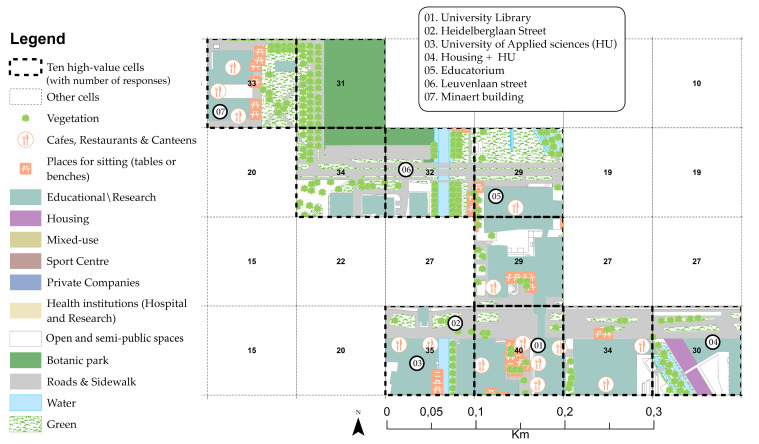
Ten high-value cells at USP.

Figure 14 and Table 9 show the results of USP high-value cells. They are scattered across two main locations: (1) in the HU and UU university library (*n* = 40 and *n* = 35); and (2) along the Minnaert building and botanic garden park (*n* = 33 and *n* = 31). Overall, all fundamental urban functions and physical features are present or close to the high-value cells. It should be noted that, as at ASP, spatial affordances for creativity at USP relate to proximity to public green areas and housing (cell 76, *n* = 30).

This descriptive analysis of the association between ‘patches’ of human perceptions and proximity to built environment features empirically tests the ‘spatial affordances for creativity’ theoretical framework [20,21,22,23,24,25,30], addressing the aforementioned research problem and research gap. Let us recall that such issues are related to the scarcity of empirical research, thus the lack of detailed information on spatial and perceptual aspects of creativity in the process of urban planning and design of campuses public spaces. Represented by the ten high-value cells of each university campus, the results support the assumption that public spaces that include or are close to a mix of land-use, activities and people can sustain creative encounters (see details in Section 2.2). 

The outcomes of this research thus form the first stage of what we hope to be an extended study on the development, use, and refinement of the quantification and association of ‘patches’ of human perceptions of creativity as well as how they can be predicted by the proximity to built environment features. This research connects to other studies in this field i.e., Brown and Reed [40], Laatikainen et al. [94], Samuelsson et al. [97] and Goodchild [99], which thus suggests that empirical data collection and the analysis of mapped experiences and perceptions can support urban design and planning decisions for multiple types of campuses and public spaces. 

### 4.3. Differences and Similarities of Creative Encounters and Public Spaces at Inner-City Campuses and Science Parks

According to the aforementioned results of the interface between perceptual data and proximity to built environment features (Section 4.2), the majority of public spaces at the Amsterdam and Utrecht campuses, located in the high-value cells, can be regarded as knowledgescapes. 

When comparing the results of inner-city campuses, we found that the high-value cells at RI campus afford creativity through a fluid interface between buildings, indoor and outdoor public spaces (see Figure 11). This is consistent with Meusburgers’ [30] studies, which suggest that such spatial fluidity can facilitate unplanned, spontaneous and cross-disciplinary discussions, and therefore creativity. On the contrary, we found that creative encounters at the OHP and Utrecht centre seem to be confined to public spaces belonging (only) to the faculty of humanities. It is also important to note that both inner-city campuses in Amsterdam and Utrecht have backyards or small gardens attached to individual buildings. Urban parks are absent in this context. It was also noted that creativity through knowledge sharing and the exchange of ideas is not necessarily dependent on public spaces, but on the spatial affordances of university buildings. 

Spaces indicated as creative clusters at SPs have a distinct dynamic compared to inner-city campuses. As mentioned in Section 4.1, users perceive SPs as having more than one main location for creative encounters. This is in line with the knowledsgescapes theory, which suggests that the spatial distribution of SPs can effectively provide ‘creative effects of heterogeneous knowledge cultures’ [50] (p. 15). Creativity seems to be less dependent on the affordances of university buildings and can occur in public spaces, since SPs provide a larger offer of public spaces than do inner-city campuses. Additionally, results of the empirical research indicate that green areas such as parks and community gardens are locations that afford creativity through knowledge sharing and exchange of ideas. The benefits of the proximity to green areas highlight that creativity somehow has a relationship with well-being. In those spaces, creativity can occur in a relaxed manner, [10,71,72] for instance when people take breaks, go for walks and interact with others, and not necessarily during working hours. 

**Figure 15:**
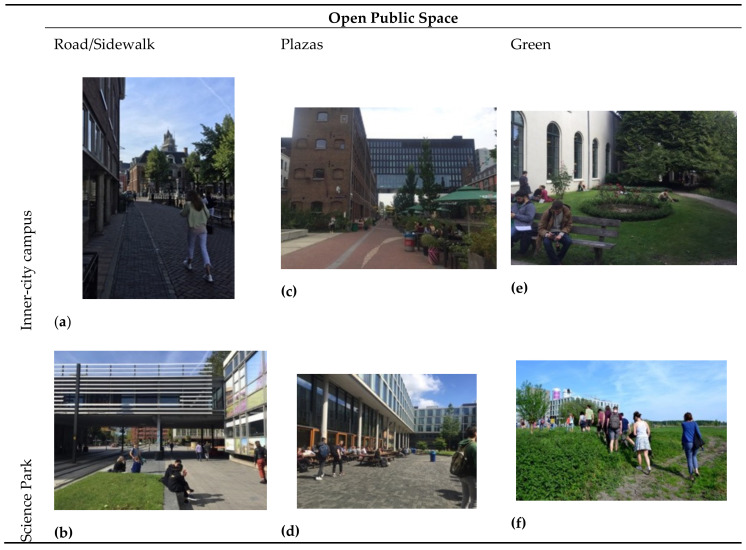
Open public spaces at inner-city campuses and SPs: (**a**) Drift street (Utrecht centre); (**b**) USP Heidelberglaan; (**c**) RI; (**d**) Outdoor area at ASP Faculty of Science; (**e**) Garden of the buildings on Drift street; (**f**) ASP Anna’s Tuin [113].

**Figure 16:**
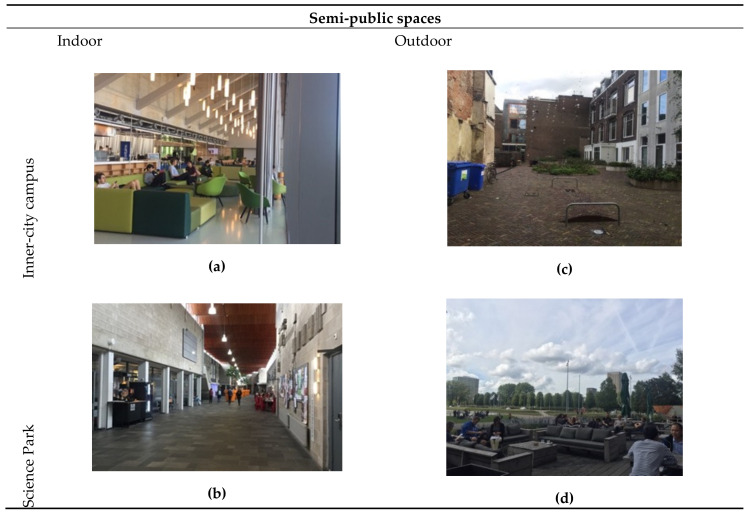
Semi-public spaces at inner-city campuses and SPs: (**a**) the ‘brug’ located at the RI; (**b**) Internal corridor at the USP Educatorium; (**c**) Backyards of buildings at inner-city Utrecht; (**d**) ASP sport centre bar/restaurant.

Figure 15 and Figure 16 illustrate open and semi-public spaces located in high-value cells at inner-city campuses and SPs. All high-value cells presented a mix of indoor, outdoor, open and semi-public public spaces. Regarding open public spaces, inner-city campuses have a very different configuration of roads, sidewalks, plazas and green areas than SPs. In inner-city campuses, roads and sidewalks are narrow and form an important part of the public space system where people can meet. Roads and sidewalks at SPs are larger, compared to the inner city, since they were originally designed exclusively for car traffic (Figure 15a,b). Inner-city campuses have what is commonly called a ‘student street’, while at SPs, because the campuses are isolated from the rest of the city, the entire area ‘belongs’ to students and those who work there.

Figure 15c,d show open public spaces at RI and ASP, demonstrating that knowledgescapes can afford creativity through the proximity to diverse amenities such as canteens, cafés, restaurants and sitting areas. This is consistent with the presented theoretical framework (see Section 2.2). Regarding green areas, gardens between buildings and classrooms are the green spaces found in inner-city campuses (Figure 15e). For SPs, green areas are open to the general public and are used for relaxation and knowledge sharing by people from multiple disciplines and organizations (Figure 15f). 

Regarding semi-public spaces (also called ‘third places’), the indoor areas of inner-city campuses and SPs are quite similar. Figure 16a,b show indoor corridors and shared spaces located at high-value cells. Photography suggests that ‘common purposes’ motivate people to spend time in those spaces, where they attain a sense of community-gathering, collective identity and symbolic ownership [61,63,64]. For outdoor semi-public spaces, however, inner-city campuses and SPs present two distinct realities. Figure 16c shows an example of the backyard of multiple university buildings at Utrecht centre (Drift street). Although this semi-public space is located within high-value cells and has the potential to facilitate a mix of tourists, day-trippers, workers and residents, the photography shows its private character that could inhibit creative encounters. This suggests that creative encounters are restricted to contacts between people from the same institution. Figure 15d is an example of a semi-private public space at an SP. Although it is a private bar and restaurant, people can circulate and interact freely.

The functional categorization of campus typologies and their public spaces is useful for highlighting differences and similarities of spatial affordances for creativity. Because the content analysis of photography was undertaken according to high-value cells, as indicated by the respondents, it is important to point out some noteworthy outcomes: (1) although SPs are spatially isolated from the rest of the city and their roads and sidewalks are arranged as an orthogonal grid, the results show that the vast offer of public spaces together with the mix of educational, research and private organizations can influence interdisciplinary encounters, and therefore creativity. This only holds true for certain locations on campus; (2) the faculty of humanities’ public spaces, in both Amsterdam and Utrecht, emerged as high-value cells for creative encounters and are located in the most diverse part of the host city; however, the content analysis of photography shows a contradictory result. The inner-city buildings and courtyards seem to be isolated from their surroundings. This was also observed in the data collection, where there is a lack of fluidity between public (buildings) and private (public spaces). 

## 5. Conclusions

We studied spatial affordances for creativity at university public spaces in the cities of Amsterdam and Utrecht. The mixed methodology comprised an analysis of VGI perceptual data and the integration of VGI data with a quantification of the built environment through proximity analyses and content analysis of photography, indicating differences and similarities between public spaces at inner-city campuses and SPs. We found that high-value grid cells, representing public spaces with a high frequency of creativity through knowledge sharing and exchange of ideas, are located within or close to multiple urban functions and physical features. The array and location of urban functions and physical features create a possibility for spatially guided creativity. We also investigated differences and similarities between open and semi-public spaces at both types of campus. Our results showed that although SPs were designed and implemented through a ‘top-down’ masterplan, they have multiple ‘creative hotspots’ compared to inner-city campuses, where creative encounters are concentrated in one or two locations. 

This study contributes to the field of campus planning and design, GIS studies, and placemaking by empirically analysing spatial affordances for creativity. The benefits of this study mainly relate to a better understanding of public spaces, which are often forgotten and neglected spaces between buildings. Through these insights, future university campuses and SPs can even more deliberately enable creativity through knowledge sharing and the exchange of ideas. We suggest that urban designers should acknowledge that public spaces must be designed in a way that affords a fluid interface between diverse organizations and disciplines together with multiple built-environment features. For creative encounters to occur, public spaces have to provide a multiple mix of active land use and activities, such as cafés, restaurants, green paths, and urban seating. This research provides evidence to support this proposition, showing that built-environment features play a fundamental role on a campus’ social dynamics, which are important for social well-being, sense of community, and mental and physical health. This applied method thus distinguishes this research from previous empirical studies.

The limitations of this study relate to the sampling design and implementation. We noticed some limitations regarding the map-based application for data collection. The shape of the polygons that represent campus public spaces differ in terms not only of user perceptions, but of user cognitive ability to read the maps and draw the polygons. These abilities can thus be affected by time, since some people need longer to become accustomed to the system. Secondly, even though the same data collection strategy was adopted for both types of campus, we received more responses at SPs that at inner-city campuses. There could be two main reasons for this: (1) the population of people based in SPs is higher and there is a broader offer of open and semi-public spaces; (2) there is a marked difference in the interface between indoor and outdoor public spaces. For inner-city campuses, people feel that they are entering a stranger’s home, whereas in SPs the interface between public and private is more fluid. 

Based on the results of a descriptive analysis, we found that proximity between diverse built- environment features does play a role in creative encounters. However, we acknowledge that future research will require a more advanced analysis using methods of spatial statistics to better understand relationships between VGI perceptual data and spatial data representing the built environment, as it was done in the research by Laatikainen, Haybatollahi and Kyttä [94] and Samuelsson et al. [97]. This would enable us to analyse the statistical significance of spatial variables relating to the values of VGI perceptual data. For the current article, we attributed the same value (or importance) for all aspects of the built environment. We thus acknowledge that future research is needed to understand the social demographics of public space users, rather than generalizing space through ‘patches’ of perceptual data. This will make it possible to explore which type of people meet in public spaces for creative purposes, based on social and spatial network theories and for different urban contexts, for instance university campus versus city centre. And finally, it would be interesting to collect and analyse qualitative data, adopting an inductive approach to analysis, to understand whether and how momentary perceptions, experiences and emotions relating to creativity are evoked by the built environment.

## Figures and Tables

**Table 1:** Summary of respondents’ socio-demographic information.

Age	Percent	Your Role in the Science Park/Campus	Percent
16–24	34.0	Company Employee	16.0
25–34	34.0	Student	43.7
35–44	18.9	University Employee	38.7
45–54	9.1	Visitor	1.6
>55	4.1	Company Employee	16.0
Total	100.0		100.0
			318 respondents

**Table 2:** Number of VGI (volunteered geographic information) responses in each Science Park and Inner-city campus

Number of Valid Responses per Campus			Number of 100 × 100 m Cells within the Study Area
Science Park/Campus	Respondents	Polygon Responses	
Amsterdam Centre	60	83	430
Utrecht Centre	49	75	192
ASP	105	166	68
USP	104	187	186

**Table 3:** Characteristics of the built environment.

Feature	Unit	What the Data Represents	Description of the Base Layer
Urban Functions			
Buildings	Polygons	For inner city: all academic and research buildings For SPs: all buildings within the institutional boundaries. For both types of campus, this includes indoor public spaces.	BGT ^1^ Top10.NL ^2^
Restaurants, canteens and cafés	Points	Points representing locations of restaurants, canteens and cafés For inner city: points within 800 m radius For SPs: points within the institutional boundaries	Top10NL OSM ^3^ observation
**Public Spaces**			
Roads and sidewalks	Polygons	Walkable roads and sidewalks	BGT
Spaces between buildings (SBB)	Polygons	Open public spaces that everyone can access Semi-public: limited access via doors/gates	BGT
Green areas	Polygons	Urban parks, gardens and areas with grass	BGT
**Physical Features**			
Sitting opportunities (tables and benches)	Points	Points representing tables and benches	BGT, OSM, observation and Amsterdam database [110]
Water features	Polygons	Canals and lakes	BGT
Vegetation	Points	Trees and small green features	BGT

^1^ Basisregistratie Grootschalige Topografie (BGT), the dataset for large-scale topography. ^2^ Top10.NL, for Dutch topographic layers. ^3^ OSM, Open Steet Map.

**Table 4:** Public space classification of Dutch campuses, based on Carr et al. [59], Oldenburg [61], Carmona [43,44], van Melik [111] and Lee [112].

Open Public Space				
Campus Type	Streets	Plazas		Green Areas
Inner-city campus	Pedestrian-oriented design, with narrow streets and no strong differentiation between roads and sidewalks. Car circulation is often limited, allowing people to walk freely.	Squares or plazas which are part of the historic development of the city centre, surrounded by high-density buildings and streets.		Commonly found in the inner city are ‘mini/pocket parks’, which are green areas bounded by buildings. Public parks can be accessible at walking or cycling distance from these campuses. Public parks are defined as ‘publicly developed and managed open spaces, often located near the centre of a city’ [59] (p. 79).
Science park (SP)	The masterplan is composed as a strictly orthogonal grid of roads. There is an orthodox separation between spaces for cars and spaces for people.	Traditional plazas are not found in science parks. They are currently being implemented.		Neighbourhood parks and community gardens may be present within SPs. Neighbourhood park: open space developed in a certain neighbourhood. Community garden: neighbourhood space designed, developed or managed by residents.
**Semi-Public Space**				
**Campus** **Type**	**Indoor**		**Outdoor**	
Inner-city campus	Consists of corridors or shared spaces, open during university hours and mostly used by people who work or study on campus. For example, community gathering spaces with tables, canteens and coffee corners.		Consists of open-air and semi-private backyards. Spaces limited by design or management policy, with access through doors or gates. These areas are mainly exclusive to university students and employees.	
Science park (SP)				

**Table 5:** Grid cells with the highest frequency of creative encounters.

Campuses	Total Polygon Responses (100%)	Responses within the Highest Cell	Location of the Highest Cell	% of Responses Based on Total Polygons
Amsterdam Inner-city	83	35	RI ^1^ campus (spaces between buildings A, B and C and E and H)	42.1%
Utrecht Inner-city	75	53	De Drift (Faculty of humanities’ classrooms and backyards)	70.6%
ASP	166	44	Indoor and outdoor spaces of the Faculty of sciences (UvA ^2^)	26.5%
USP	187	40	USP University Library and Faculty of social sciences	21.3%

^1^ RoeterseIland Campus (RI). ^2^ University of Amsterdam (UvA).

**Table 6:** Distance between VGI data and built-environment features. Amsterdam inner-city campuses.

Cell ID	High VGI Values (*n*)	Distance to:															
		Urban Functions										Physical Features					
		Campus Buildings		Restaurant, Café or Canteen		Road and/or Sidewalk		Spaces between Buildings (SBB)		Green		Tables and Benches		Water		Vegetation	
		CWC ^1^	DN ^3^	CWC	DN	CWC	DN	CWC	DN	CWC	DN	CWC	DN	CWC	DN	CWC	DN
97 (RI)	35	x ^2^		x		x		x			7	x		x		x	
125 (RI)	32	x		x		x		x		x		x		x		x	
96 (RI)	24	x		x		x		x			17	x		x		x	
124 (RI)	21	x		x		x		x		x			8	x		x	
126 (RI)	20	x			8	x		x		x		x		x		x	
271(OHP)	19	x			15	x		x			36		35	x		x	
272(OHP)	19	x			3	x		x		x		x		x		x	
273(OHP)	18	x			35	x		x			38		27	x		x	
294(OHP)	18	x		x		x		x			65		7	x		x	
295(OHP)	18	x		x		x		x			82		35	x		x	

^1^ CWC = Contain Within the 100 × 100 m Cell., ^2^ “x” represents a cell that the feature contain within the 100 × 100 m cell., ^3^ DN = Distance to Nearest (in meters).

**Table 7:** Distances between VGI data and built-environment features at ASP.

Cell ID	High VGI Values (*n*)	Distance to:															
		Urban Functions										Physical Features					
		Campus Buildings		Restaurant, Café or Canteen		Road and/or Sidewalk		Spaces between Buildings (SBB)		Green		Tables and Benches		Water		Vegetation	
		CWC ^1^	DN ^3^	CWC	DN	CWC	DN	CWC	DN	CWC	DN	CWC	DN	CWC	DN	CWC	DN
22	44	x ^2^		x		x		x		x		x		x		x	
12	36	x		x		x		x		x			2	x		x	
29	34	x			2	x		x		x			17	x		x	
21	33	x			10	x		x		x		x			5	x	
14	31	x			59	x		x		x			35	x		x	
30	31	x		x		x		x		x		x		x		x	
33	31	x		x		x		x		x		x		x			20
20	20	x		x		x		x		x		x		x			1
13	26	x			60	x		x		x			35	x		x	
18	24	x		x		x		x		x			17	x			10

^1^ CWC = Contain Within the 100 × 100m Cell., ^2^ “x” represents a cell that the feature contain within the 100x100m cell., ^3^ DN = Distance to Nearest (in meters).

**Table 8:** Distances between VGI data and built-environment features at UU inner-city campus.

Cell ID	High VGI Values (*n*)	Distance to:															
		Urban Functions										Physical Features					
		Campus Buildings		Restaurant Café or Canteen		Road and/or Sidewalk		Spaces between Buildings (SBB)		Green		Tables and Benches		Water		Vegetation	
		CWC ^1^	DN ^3^	CWC	DN	CWC	DN	CWC	DN	CWC	DN	CWC	DN	CWC	DN	CWC	DN
146	53	x ^2^			25	x		x			34	x			12	x	
145	47	x			24	x		x		x			9	x		x	
163	46	x		x		x		x			23	x			5		1
162	38	x		x		x		x		x		x		x		x	
127	30	x		x		x			20	x		x		x		x	
128	29	x		x		x		x			22		39		18		9
75	17	x		x		x		x		x		x			14	x	
109	17		25	x		x		x			4		8	x		x	
110	17	x			32	x		x		x			18	x		x	
126	17	x		x		x		x		x		x			48	x	

^1^ CWC = Contain Within the 100 × 100m Cell., ^2^ “x” represents a cell that the feature contain within the 100x100m cell., ^3^ DN = Distance to Nearest (in meters).

**Table 9:** Distances between VGI data and built-environment features USP.

Cell ID	Highest VGI Values	Distance to:															
		Urban Functions										Physical Features					
		Campus Buildings		Restaurant Café or Canteen		Road and/or Sidewalk		Spaces between Buildings (SBB)		Green		Tables and Benches		Water		Vegetation	
		CWC ^1^	DN ^3^	CWC	DN	CWC	DN	CWC	DN	CWC	DN	CWC	DN	CWC	DN	CWC	DN
74	40	x ^2^		x		x		x		x		x			21	x	
73	35	x		x		x		x		x		x		x		x	
75	34	x		x		x		x		x		x			2	x	
104	34	x			38	x		x		x			16		54	x	
119	33	x		x		x		x		x		x			5	x	
105	32	x		x		x		x		x			30	x		x	
120	31	x			21	x		x		x			46		1.45	x	
76	30	x			11	x		x		x			11	x		x	
90	29	x		x		x		x		x		x			20	x	
106	29	x		x		x		x		x		x		x		x	

^1^ CWC = Contain Within the 100 × 100m Cell., ^2^ “x” represents a cell that the feature contain within the 100x100m cell., ^3^ DN = Distance to Nearest (in meters).
